# Empowering families to take on a palliative caregiver role for patients with cancer in India: Persistent challenges and promising strategies

**DOI:** 10.1371/journal.pone.0274770

**Published:** 2022-09-16

**Authors:** Soraya Fereydooni, Karl A. Lorenz, Archana Ganesh, Anchal Satija, Odette Spruijt, Sushma Bhatnagar, Raziel C. Gamboa, Nainwant Singh, Karleen F. Giannitrapani

**Affiliations:** 1 Center for Innovation to Implementation (Ci2i), VA Palo Alto Health Care System, Palo Alto, California, United States of America; 2 Yale School of Medicine, New Haven, Connecticut, United States of America; 3 Stanford University School of Medicine, Palo Alto, California, United States of America; 4 All India Institute of Medical Science (AIIMS), New Delhi, India; 5 Peter MacCallum Cancer Center, Melbourne, Victoria, Australia; Universitat Luzern, SWITZERLAND

## Abstract

**Background:**

The population of patients with cancer requiring palliative care (PC) is on the rise in India. Family caregivers will be essential members of the care team in the provision of PC.

**Objective:**

We aimed to characterize provider perspectives of the challenges that Indian families face in taking on a palliative caregiving role.

**Method:**

Data for this analysis came from an evaluation of the PC-PAICE project, a series of quality improvement interventions for PC in India. We conducted 44 in-depth semi-structured interviews with organizational leaders and clinical team members at seven geographically and structurally diverse settings. Through thematic content analysis, themes relating to the caregivers’ role were identified using a combination of deductive and inductive approaches.

**Result:**

Contextual challenges to taking up the PC caregiving role included family members’ limited knowledge about PC and cancer, the necessity of training for caregiving responsibilities, and cultural preferences for pursuing curative treatments over palliative ones. Some logistical challenges include financial, time, and mental health limitations that family caregivers may encounter when navigating the expectations of taking on the caregiving role. Strategies to facilitate family buy-in for PC provision include adopting a family care model, connecting them to services provided by Non-Governmental Organizations, leveraging volunteers and social workers to foster PC awareness and training, and responding specifically to family’s requests.

**Conclusion:**

Understanding and addressing the various challenges that families face in adopting the caregiver role are essential steps in the provision and expansion of PC in India. Locally initiated quality improvement projects can be a way to address these challenges based on the context.

## Introduction

Palliative care (PC) needs for non-communicable diseases, particularly cancer, is on the rise in India [1]. Accounting for 63% of all deaths, non-communicable diseases are the prevailing cause of disease burden with cancer as the leading cause of death (9%) [2, 3]. It is estimated that 1 in 9 Indians will develop cancer during their lifetime and 1 in 7 will pass away [2]. Cancer is mostly diagnosed at a late stage in India [4]. As such, pain and symptom management as a part of PC provision is an apt treatment plan [4]. The chronic nature of cancer demands involvement of the home and the informal caregiver as a part of the care [5].

In India, families play a significant role in end-of-life care and caregiving to family members with illness [6]. Informal caregiving to patients may fall to the family for many different reasons including tradition, dearth of formal care, or lack of financial resources to afford them [7]. As family is the most important informal social protection, caregiving to ill family members is an expected responsibility in India [8]. Professional care and institutional facilities for end-of-life care are inaccessible in many areas of India especially low-income settings [9]. Extant community-based initiatives which aim to extend PC access to the poorest are home-based [10]. As such, family caregivers are an integral part of the existence and expansion of programs such as Neighborhood Network in PC. Similar to community health workers and promotors, this is a community-led initiative in south India to provide home-based palliative care and improve access to palliative care services [10, 11].

Caregiving to an ill patient includes a range of supportive activities which may incur personal challenges and costs in the caregivers’ lives [6]. Some of these supportive activities include assisting with emotional wellbeing and activities of daily living, fulfilling treatment recommendations, and aiding in critical medical decision-making [12]. Caregivers promote continuity of care in the hospital and often actively facilitate care with health care teams [13]. Their unpaid labor is cost saving for the healthcare system while incurring profound personal burdens [14, 15]. These may include disruptions in education or career plans, role conflicts, isolation, and a range of chronic physical and mental illnesses [15]. The chronic stress of being a caregiver to a cancer patient may even predispose caregivers to increased inflammatory responses and morbidities such as systemic inflammation, immune system dysfunction, hypertension, and cardiovascular disease [16]. Despite this fact, evidence-based interventions to alleviate burdens of caregiving for cancer patients have been limited [17].

Treating family caregivers as members of the care team and responding to their needs is essential to high-quality palliative and end-of-life care [18, 19]. Promoting Access and International Cancer Experience (PC-PAICE) was an international collaborative to support quality improvement in palliative care in India [20]. Among healthcare providers and organizational leaders we interviewed to evaluate the effectiveness of PC-PAICE’s initial cohort, caregivers were consistently mentioned as key to the success of home-based palliative care. The mention of caregivers as a factor in the quality improvement (QI) projects prompted a secondary analysis of the PC-PAICE interviews, exploring themes related to end-of-life caregiving. Our goal was to understand what stakeholders perceived as barriers to taking up a caregiving role and what were some approaches to address these barriers.

## Methods

This study was approved by both the Stanford University Institutional review board (IRB– 42633) and the Institute Ethics Committee of the All India Institute of Medical Sciences, New Delhi (IEC-572/03/11.2017, RP-41/2017).

### Setting

This is a secondary analysis of data collected as part of a greater effort to evaluate the implementation of the PC-PAICE initiative, a multi-site effort to improve palliative care delivery in India via locally initiated quality improvement projects. The seven sites selected for the PC-PAICE project were geographically diverse (multiple states) and structurally diverse including PC clinics, large academic institutions, and oncology hospitals. Additional details are available on PC-PAICE and evaluation of the initial cohort [20].

### Approach overview

We conducted in-depth semi-structured interviews at the seven geographically diverse sites of the PC-PAICE initiative. The interview guide, informed by the consolidated framework for implementation research, was pilot tested and solicited feedback about participation in the PC-PAICE program and experiences of implementation. We employed a quota sampling approach to capture the perspectives of diverse stakeholders including organizational leaders, clinic leaders, and clinical team members (medical doctors, medical residents, nurses, and social workers) [21].

A PC-PAICE champion, who is a clinical leader and an organizational partner, facilitated the resources to participate in PC-PAICE, undertake the QI project, and provide introductions to important local stakeholders. A member of our team then contacted the interviewee via email and telephone to request participation. They were informed that 2 Indian researchers from the PC-PAICE program will be interviewing them on their participation experience. There were no dropouts or refusals. The site champion ultimately coordinated interview schedules, site visits, and space for the interviews. In the two cases that geographical flooding prohibited in-person interviews, we conducted a total of 13 interviews from both sites on the phone. Only the researcher and interviewees were present for the interviews. All participation was voluntary and completed with written informed consent which highlighted the purpose of the study, the participants’ right to terminate the interview, and informed them that the interviews were being recorded. Most interviews (43/44) were audio-recorded and one participant requested written notes only, though all 44 interviews were used for analysis. All audio-recorded interviews were transcribed verbatim by AG and AS. The transcripts were not returned to the interviewees for validation.

### Data collection

Our team was comprised of three PC physicians (KL, OS, SB), one social scientist with expertise in qualitative methodologies (KG), and multiple analysts. All researchers except KL were female. KG did in person training for two analysts based in India (AG, AS) on interviewing techniques prior to commencing data collection and accompanied them on interviews at initial sites for quality assurance. All interviews were then attended by both analysts with one conducting the interview and the second taking detailed notes. The 43 interviews that were audio-recorded were transcribed and de-identified.

### Data analysis

Analysis occurred in multiple steps combining inductive and deductive approaches. First, three team members (KG, AG, AS) open-coded one transcript and met to consolidate the open codes into a code list with code definitions (inductive). The analysts piloted the code list on three other transcripts separately and reached consensus around codes and their definitions. This code list was then applied to all remaining transcripts by an analyst and reviewed by a second (deductive). The analysis was conducted via Atlas. ti Version 8 [22]. Bi-weekly team meetings were held over four months to manage coding discrepancies and come to a consensus around code application. One of the codes in this overall code list was the Role of Caregivers on the PC-PAICE team. There were 101 mentions of the caregiver role. All the output from the Caregiver code was then used as the total data set for thematic content analysis [23]. KG and SF took a constant comparison approach to the caregiver output to identify themes through iterative review until consensus was reached (inductive) [24]. The themes were given a code name and applied to other caregiver outputs (deductive).

## Results

We identified three major themes on barriers and solutions to taking up the palliative caregiver role in India.

### Theme 1: There are key cultural and factual knowledge barriers to family members buy-in the palliative caregiving role

#### Subtheme 1.a: Family caregiver knowledge about cancer and PC

Community-wide misconceptions or lack of knowledge about PC and cancer were barriers to family engagement with palliative caregiving. For instance, the misconception that cancer is contagious sometimes led families to abandon the role of caregiving altogether to avoid stigma from the broader community. For example, a participant mentioned “if somebody’s wife has this disease [cancer], then their husband’s avoid them or leave them in their parents’ house and visit them once in a while.” Other times, misconceptions about PC led to resistance from the family and hindrance of care. Some examples included lack of cooperation with providers to locate the patient’s house for home-based PC, asking the providers to run errands, perceiving a provider’s care recommendation as meddling in family affairs, and sometimes even having aggressive encounters with the providers: “*Her son came and shouted at me*. *Who are you*? *Why you came here*? *What is the benefit you are going to get*?” (Clinical team member) As such, providers felt the need to “*educate the society in general about dignity of life and the issues of the importance of PC and why at a certain stage you have to move on from definitive care to PC*.*” (Organization lead)*

This unawareness about the role and purpose of PC is not only inherently present in the broader community but is also exacerbated by the absence of communication from oncologists. For example, one PC provider described how families are frequently referred to PC without any explanation. In these situations, the PC provider is responsible for breaking the emotionally charged news about the patient’s prognosis to the patient and their family, as well as explaining the PC process, and starting care. Due to absence of communication, families may perceive PC as a step in the curative treatment process. As a result of this false expectation “*their [family and patient’s] hope gets destroyed in one minute and the patient gets a big shock*,*”* (Clinical team member) leaving them angry with the PC team and pursuing discharge of the patient “*within two hours of patient admission*.” (Clinical team member).

#### Subtheme 1.b: Caregiver preference for curative treatment

Initially, providers perceived that providing PC “*will be easier here in India because most of us believe in the Hindu philosophy… we accept fate… so convincing them about the terminal care for palliative care may be relatively easy*.” (Organization lead) However, it was reported by multiple providers that “*delivering palliative care in India is very difficult” even when* “*the oncologist has explained that nothing can be done*,*”* the family “*drag[s] the patient till the last moment*.” (Clinical Team Member) This phenomenon was especially common among educated and wealthy people who perceived “*that palliative care is almost like giving up*,” leading to “*a lot of families [going] bankrupt in the process because they sell their properties for treatment*.” (Clinical lead) However, one provider cautioned that “*you cannot generaliz*e” this perception, and “*it varies from patient to patient*.” (Clinical Team Member) Some families that “*have almost tried everything they might understand that an aggressive management might not be helpful and they might understand that the palliative care is more important even without our communication*.” (Clinical Team Member).

#### Subtheme 1.c: Lack of family training on how to fulfill caregiving responsibilities

Providers from multiple sites highlighted that while “*family can be a huge support*” (Clinical lead) in India, they are often expected to take on the role of caregiver without prior training. Providers noted that some of these families “*don’t know how [to] even [have] a proper hygiene*. *Family doesn’t know it’s important to take a proper brushing every day*, *it’s important to take a bath every day*.” (Clinical Team Member) Given the gap between where the families were starting from and where they needed to be to assume a caregiver role, providers highlighted a significant need for “*qualified*” people to provide training:

“*Mainly cancer you need adequate staff*, *you need adequate volunteers and staff… in the sense qualified staff*, *I mean… for e*.*g*. *if you go to a patient who is totally bedridden with cancer and who can’t eat or drink*, *you have to teach them and the relatives on how to put a drip you know under the skin… that sort of a thing… and how to do an enema… and how to do a manual evacuation of the rectum… all this you need particular staff for it…”*

(Clinical Team Member).

Training family members to take on such responsibilities took investment on the part of the providers and system. However, often such human resources and provider time to fill this family training gap were lacking.

### Theme 2: Family caregivers in India may face an overwhelming tradeoff between caregiving and financial and time demands related to work

Providers note that some poorer families may feel unable taking on care for a patient because of financial challenges, time constraints, and the obligation to work outside the house: “*family members don’t look upon the patient because of financial reasons… and mostly the family members go to the work in the morning*.” (Clinical Team Member) The trade-off of holding an income-earning job coupled with the sentiment that “*a fellow is destined to die*, *so let him die… why make an effort to spend time and money on this patient*” (Clinical Lead) makes taking on caregiving a difficult proposition. Providers noted that especially those families who live far away from the hospital, may not bring the patient for care when they should or take them home when their treatment is over. Providers also observe inter-family conflict where one child feels like they are “*doing a lot and other children are not doing*.” (Clinical Team Member) In these families, caregiving is sometimes bestowed upon a “*caregiver who is untrained and unmotivated*,” (Clinical Team Member) leading to poor care for the patient: “*you [PC provider] give them [family member] advice*, *medication*, *the time dosage*, *frequency everything… then they are not adhered to… many times when you count the tablets of morphine they are having*, *you know that they are not giving them as per their dose*.” (Clinical Team Member).

One provider also empathized with waning energy in caring for the patient as caregivers are generally there “for the patient 24*7, *and they do not have their personal life*,*”* (Clinical Team Member) and the prospect of focusing on the patient who is going to die soon represents huge mental health burden. In these situations, despite receiving support from the volunteers, some caregivers “*vanish*” and “*the patient will be alone at home*, *nobody to take care*,*”* (Clinical Team Member) leading to not taking medication and poor hygiene, etc. While the use of hospices is less popular in India because of the family-centered culture, sometimes the caregivers have no choice but to leave these patients in the hospice, which patients like better “*because there is someone to take care of them*.*”* (Clinical Team Member) One provider explains how reliance on the family for caregiving in India can be a double-edged sword:

“*our geriatric population is increasing… We cannot expect the family to look after everyone…that can only be an added support*. *We have to start growing out of box in thinking; that it’s not wrong to be looked after in a in a facility and having family support with the facility would probably be a very nice thing*.”

(Clinical Lead)

Simultaneously, in other families, there will be personal and cultural pressure to serve as the primary caregiver for the patient. In some families this ideology is so strong that “*the family members think that it is a sin on them… if they don’t take care of the patient then they have done some mistake*.*”* (Clinical Team Member) Family caregivers in India may feel they face a lose-lose proposition under the pressure of cultural and financial constraints.

### Theme 3: Grassroot strategies can facilitate family buy-in for PC provision

#### Subtheme 3.a: Adopting a family care model to train and empower the caregivers

Providers emphasized the importance of *adopting a “family care model*” rather than solely focusing on the patient. (Clinical lead) In this model there is “*counselling for the whole family on what to do and what not to do*.” (Clinical team member) Some family caregivers benefited from a broad orientation to caregiving such as providing training on specific tasks like putting on clothes, taking care of heavy bleeding, wound dressing, putting drips under the skin, giving medications, administering enemas, and giving fluids after patients are discharged. They also mentioned facilitating specific patient goals and last wishes such as expression of intimacy.

“*We try to counsel the ladies as well as the patient that maintain hygiene condition*. *We need to think of the patient…maybe it’s his last wish*, *so*, *we should not deny his wish*. *On the other hand*, *we talk about the wife’s concerns…concerns like malodor*, *untidy feeling can be taken care of by maintaining hygiene and covering the cancerous/untidy part*. *If the wife takes care of such things*, *then it can be possible*. *If there is so much love between the two*, *then that should be expressed also.”*

(Clinical team member)

#### Subtheme 3.b: Raising acceptance of PC through social workers, volunteers, and community networks

Providers talked about the importance of activating families on an individual level and the broader community through volunteers in raising acceptance of PC. In the beginning of care, social workers would sit down with the families “*to see what they understand and what they don’t understand… what are their expectations*.” (Clinical team member) For example, “*if something is there like their expectation is the ICU care*” they corrected the misconception. At one site where the family “*left against medical advice*, *absconded from treatment”* social workers intervened and talked to the family to “*break the barriers and convince them to come back*.” (Clinical lead).

Buy-in from one family further had ripple effects throughout the community via the “*word of mouth*” effect:

“*They will motivate somebody else and then somebody else and again somebody else in the same city or town… 98% of our reference are actually from word of mouth…* “*I went to that hospital and [the patient] died very peacefully… so you can also take them there.*””

(Organization lead)

However, solely focusing on educating the caregiver was usually not enough, as caregivers were influenced by the broader community and society:

“*It does not go into their mind because I am just single doctor who is explaining in a proper way but he [caregiver] listens to all the surrounding area*, *his relatives etc*. *etc*. *and they will think that you should have took him to that hospital [for curative treatment]… So*, *it will be very difficult for one person to clarify the thing*.*”*

(Clinical team member)

To provide awareness and resources on a larger scale, community initiatives such as the Neighborhood Network in Palliative Care have been developed in which volunteers get trained “w*hat palliative care is all about and how important that is in our community… and how they would assess the people who need PC*” [10]. (Clinical team member) There are also other initiatives such as “*free kitchen in hospice*”, “*fundraising activities*”, (Organization lead) and donations which are partially fueled by previous caregivers who have come into contact with PC:

“*We asked them [volunteers] what motivated you to do this… and the initiation is that they had somebody in the family who actually suffered very badly when they were terminally ill and nobody was there to guide them…so that is the personal tragedy which motivated them…”*

(Organization lead)

#### Subtheme 3.c: Responding to the family caregiver’s requests

Providers noted the importance of responding to broader societal pressure and family preference in getting the caregiver’s buy-in for the provision of PC. For instance, some caregivers had a hard time accepting home-based PC due to the social stigma associated with cancer:

“…cancer is sometimes they think it is contagious… the patients and the relatives don’t tell their neighbors that somebody is down with cancer.. so they say ‘no no.. you have to come in secret.. don’t come when they are seeing…”

In response to this request, PC providers had tried various specific strategies such as parking far away from the house, dressing in non-medical clothing, and bringing along a social worker during home care visits to mitigate any issues with the family and the neighbors.

Disclosure of prognosis was another avenue where families had specific requests. In many cases, “*the family members come just before handing over the patient and they just say that ‘the patient does not know about the diagnosis and the actual situation… and so we would like your doctors to actually not talk about it*.” (Clinical team member) This family request stemmed from the fear that the patient would suffer from depression and stop fighting for their health. In these situations, even though providers found this ethically challenging, they usually complied with the caregivers “*because they don’t want to cause any problems”* as “*they [caregivers] are the people who are caring for the patient 24 hour*s” and they required “*the confidence of the caretaker before talking to the patient*.” (Clinical lead).

Some centers have tried to counsel the family, “*trying to find out the reasons why they [caregivers] don’t want us to disclose it [the prognosis] to the patient*” (Clinical team member) and “*taking proactive steps to resolve such collusion*” (Organization lead) by explaining to the family that they were not “*destroying patient’s hope*,” (Clinical lead) rather gradually breaking the news to them using appropriate communication skills. Other centers have adopted QI projects that along with the patient’s goals of care, documents family and patient preferences for disclosure of diagnosis for the care team.

#### Subtheme 3.d: Providing material resources such as financial relief and mental health care for the caregiver

Providers emphasized the importance of following up with and supporting caregivers who didn’t bring patients in for consistent care. Non-Governmental Organizations and smaller departments provided support in the forms of financial assistance and mental health care support.

Financial support was usually offered to families who were poor and lived far away from the centers, in the form of free or subsidized medicine, food, transportation, or lodging near the PC center. In families where the patients used to be the breadwinner, the volunteers also “*conduct rehabilitation camps where they teach the patient or the family to make some products”* such as umbrellas, soap, and clothes, “*and there is a college who even supports selling those products in the campus*.” (Clinical lead).

Mental health care support was offered to families through facilitating “*entertainment program of singing and dancing*,*”* (Clinical team member) burial ceremonies on behalf of poor families, or participation in other ceremonies. For instance, one provider mentioned hosting a procession called “*Ashadi Ekadashi*” for the patients and their caregivers in the center:

“*We arrange a small procession or Yatra here [center] itself… so it starts from the auditorium and it goes to each and every ward… so these are also actually become the diversion activities… and ultimately it is quite comforting for them…”*

(Clinical team member)

Providers also took over for caregivers because they recognized the challenge that caregivers “*do not have their personal life…[and] that balancing becomes difficult… [as] this patient is eventually going to die*, *so what is [the caregiver] left with*?” (Clinical team member). Providing emotional support for the caregiver was often the “*reason why they come back*” because the PC providers “*give them a good ear and listen to their problems*.” (Clinical lead) This respect for the caregiver’s emotional wellbeing was approached systematically in some centers through “*almost weekly [meetings] with the caregivers*” where the staff asked for feedback. (Organization lead).

## Discussion

Considering the cultural and economic context of India, family caregivers play an integral role in the provision of quality PC for cancer patients. As such, respecting and responding to their emotional, informational, cultural, and financial needs are necessary to their inclusion in the care team. Our study from a geographically and culturally heterogeneous sample in India specifically characterizes challenges faced by caregivers in the provision of PC from the provider’s perspective. The study highlights how grassroots solutions were helpful in empowering and including caregivers. Challenges included stigma, lack of awareness, financial tradeoffs, time and emotional constraints, lack of training, and balancing cultural preferences. Some solutions include forms that document caregiver preferences, incorporation of social workers, material assistance, training caregivers, and promoting broader awareness of PC through Neighborhood Network in Palliative Care [10].

Providers perceived that many families felt unprepared to take on palliative caregiving roles. This is not surprising considering that caregiving is not perceived as a separate role from other familial obligations, which means that there’s less recognition around the necessity of specialized training for the role [8]. While volunteers currently fulfill some of this need for training, gaps remain. Emerging solutions from other contexts include distribution of basic skills training on CDs or online, education on informal caregiving for Latinx caregivers via bilingual soap operas, and use of kits eases acute symptom management by color coding a symptom and its corresponding medication [25–29]. These efforts have been found to improve caregiver self-efficacy, reduce caregiver distress and anxiety, improve patients’ quality of life, and reduce the number of hospital visits [30]. These broad-based approaches can complement existing solutions in India such as one on one training from volunteers who can further support caregivers in fulfilling specific patient goals.

Another deterring factor for families to take up the caregiving role is the stigma associated with cancer and PC. Stigma is a prevalent phenomenon in India and is associated with many illnesses such as HIV/AIDS, diabetes, various mental illnesses, cancer, and tuberculosis [31–36]. Specifically, cancer-related stigma has been traced back to fears of disease transmission, and the belief that the patient’s actions are somehow responsible for their developing cancer [33, 37–39]. To reduce stigma, interventions such as mass media campaigns, the inclusion of PC as part of regular cancer care, and other community-based initiatives that encourage caregivers and patients to share their experiences have been adopted [40–43]. Similarly, providers in our study emphasized the importance of both individualized and community interventions, such as counseling and Neighborhood Network in Palliative Care, in raising awareness and combating stigma [10]. They further discussed strategies to collaborate with the caregiver, like hiding their vans and not dressing in professional clothing, to keep PC provision private from their broader community.

As reported by our participants, balancing the cultural preference for families to serve as informal caregivers, the limited alternative institutional facilities, and the economic tradeoff that comes with caregiving were dilemmas faced by many families. To reduce this burden, many low-middle-income countries have introduced social protection programs that provide cash and other benefits such as staple foods, subsidized utilities, or income transfers to older adults [44]. Similar programs were also mentioned in our study, though the efficacy and sustainability of these programs remain uncertain [44]. Some sustainable strategies mentioned in our study include training caregivers in different income-generating skills such as umbrella and soap making. Other suggested solution in facilitating economic resiliency for families is furthering families’ training in caregiving through formalized programs to earn certificates and reenter the workforce [45]. An added benefit is the introduction of a large number of skilled caregivers in the delivery of end-of-life care, especially to underserved areas [46]. Indeed, our findings confirm the importance and burden of the caregiving role on families and suggest some strategies to address the subsequent need for social support and welfare programs that could complement the Essential Package of Palliative Care [47]. The Essential Package contains the necessary components for pain relief and palliative care provision.

One novel positive example of using a site-initiated QI project to respond to caregivers’ needs included addressing the family’s preferences for disclosing the prognosis to the patient. Providers recognized the importance of respecting caregivers’ needs as members of the care team. To keep all the other team members informed, providers at one site documented caregiver preferences for disclosure of prognosis in the patient chart. In addition, they tried fulfilling those preferences throughout the care process unless the progression of care necessitated disclosure to the patient. When disclosing the diagnosis to the patient was needed, trained social workers would counsel the family, explaining the reason and process of disclosure. While the ethically challenging problem of provider-family collusion in withholding information to the patient is not a unique finding, the use of a systematic approach (e.g. engagement in QI) to account for family-caregivers as a part of the care team is a finding that is worth exploring further [48].

Caregiving burden is a well-known concept and includes emotional, interpersonal, physical, financial, and social consequences [49]. The types of challenges raised by our participants also fit within these categories identified in the broader literature placed in the US. However, there are noteworthy differences in their presentation and solutions compared to the west. For example, while caregiving can detract from job participation, enlisting aid from an outside facility is culturally more accepted in the west compared to India [50–52]. Furthermore, cancer is stigmatized in India and PC centers work with the family to protect their privacy during home-based services. While hiding the diagnosis from the patient is unacceptable in the west, in India this request is usually fulfilled to encourage buy-in from family members [52]. These examples illuminate that though the types of caregivers remain consistent burden across contexts, the nuances of these problems vary, demanding ground-up solutions facilitated through QI methods.

This study can be considered in light of the following limitations. This is a secondary analysis of the data collected as part of a greater effort to evaluate the implementation of PC-PAICE [53]. However, the themes surrounding caregiver buy-in were so strongly represented in our interviews that they warranted the secondary analysis presented here. If it had been the original intent of the study, we would have included additional interview probes about the role of caregivers. Moreover, the participants in this study were not directly caregivers, rather organizational leaders, clinicians, and team members who discussed the importance of caregiver roles. Future study efforts should directly explore caregiver and patient perceptions of PC needs, caregiving challenges, and potential corresponding solutions. India is home to a vast number of disparate cultures, and our study does not provide in-depth or specific insights into any Indian cultural group; however, our seven sites were widely geographically dispersed and therefore the dominant themes represent prevalent, if not group-specific issues in India.

## Conclusion

Family caregivers play an essential role in the care team and provision of PC for patients with cancer. A first step in facilitating their role in PC is characterizing the financial, emotional, cultural, and limited information challenges that caregivers face in assuming the PC caregiving role. Addressing the various needs of family palliative caregivers is a big undertaking. Future work can leverage locally initiated QI projects to address these challenges appropriately based on the context.

## Supporting information

S1 File Minimal data set. Complete quotations used in the manuscript.
(DOCX)

S2 File PLOS’ questionnaire. (DOCX)
