# Supplementary material for: Empowering families to take on a palliative caregiver role for patients with cancer in India: Persistent challenges and promising strategies
Source: PLoS One. 2022 Sep 16;17(9):e0274770. doi: 10.1371/journal.pone.0274770 (PMC9481001; doi:10.1371/journal.pone.0274770)
Supplement: S1 File — Complete quotations used in the manuscript. (DOCX) [file pone.0274770.s001.docx]

Table 1. Complete quotation used in the manuscript

| **Subtheme 1.a: Family caregiver knowledge about cancer and PC** | |
| --- | --- |
| “ well you know the patient background, their economic status, their financial status, their family backgrounds… for a lot of them..  gets emotional and begins crying….  Because of their disease and pain, they are left alone and not cared for.. neighbours look down upon them, feel disgusted and avoid them. If somebody’s wife has this disease, then their husband’s avoid them or leave them in their parents house and visit them once in a while…  gets emotional and begins crying again….  So there is a lot in home care service you know.. so we identify all this and provide medicines also if required..” | |
| “So that is another very important thing. Currently, unfortunately awareness among patients is also very less.. and therefore there is a need to even educate the society in general about dignity of life and the issues of the importance of palliative care and why at a certain stage you have to move on from definitive care to palliative care.. and other issues in terms of not only dignity in life, but dignity in death.. and giving that patient the sort of care that they deserve during the last stage of his life..” | |
| “madam. So, I just told you that patients are referred here for morphine. Similarly, we get referrals for only palliative care, symptom care, best supportive care, like, they write for takeover to palliative care. But they do not know what is being done in palliative care? Here, we do not give curable treatment, do not give chemo. Palliative RT and CT is not thoroughly given. They don’t tell them that in palliative care, mainly pain treatment is provided. This a practice of not only oncology side people in Site-A but also from another hospital. They say, ‘Go for palliative care. Your treatment is complete here and start the treatment there (at Site-A)’. But they don’t tell what treatment will be provided at our department is not told to the patient and the attender. They ask us if this treatment will reduce the cancer size. When we tell them that ‘We are not from oncology side but from palliative care’, they get angry with us. They say ‘The condition of the patient is very bad. He is very sick. You do something. Put him on oxygen. Give him fluids.’ They pressurise us for these things. ‘Admit him in ICU’, which is not possible in palliative care. It is difficult for them to understand all this.  R1: You mean to say that other doctors do not give full information to the patients about the palliative care. They refer patients to you without explaining them what exactly is their disease status and then when you tell the truth to the patient that ‘Cure is not possible’, then they get angry with you…What do the relatives and the patients need to do at other times? Other doctors have referred them over here. Patients say other things when they come here. So, they don’t understand what to do. That’s why they get angry with us. The patient and attender are in problem. Till now, they believed that the patient is curable, they have some hope. Their hope gets destroyed in one minute after talking to us and then patient gets big shock.” | |
| “ Many times, we see that within two hours of patient admission, they take the discharge from here, saying that ‘No, no, we don’t want our patient to stay here’. They are not happy because in palliative care there are only stable things. Patient is going, okay, stability is there. No hurry. We have no ICU here. There is no hustle bustle here. Doctors are not rushing here in panic. So, what is this happening? Such challenges are observed. This is not observed with the corporate people. But with INI hospital, it is only for oncology patients and they concentrate mainly on curative treatment.” | |
| **Subtheme 1.b: Caregiver preference for curative treatment** | |
| “ Culturally I think it will be easier here in India because most of us believe in the Hindu philosophy.. we accept fate, families.. so convincing them about the terminal care for palliative care may be relatively easy..” | |
| “ It’s, to be frank, delivering palliative care in India is very difficult because..in Indian context the family...completely thinks to drag the patient till the last moment. Whereas in the foreign overseas, we will deliver this thing that ‘Yes, he’s been suffering from so and so and he may not be living for so and so days.’ So, the last part of the treatment, okay, doctor you don’t give up, just let him live like that. Let him be happy. Let him spend time with the family. Everything, they, they will be like much more comfortable in doing that. When it comes to the Indian con..Indian side they ask, even if the patient is dying in another 10-15 minutes, ‘Doctor why aren’t you doing anything? Why don’t you provide? Why don’t you do give an injection, revive him back and make him’..like we tell them, if I, I revive him back, he will suffer for more couple of days and me speaking like that they won’t take it properly and that can be a conflict with the doctors sometimes and for delivering it in a proper way is also important and after delivering it also, they will get in their mind but....one..like when patient is also dying, they accept- yes, he is dying. But the small hope or guilt will be there and they are feeling like ‘I couldn’t do till the last’. That is there.” | |
| “Difficult to..still even the girl which I told, I have explained the mother saying that nothing can be done. Even the oncologist has explained that nothing can be done. Today after seeing me, she is asking it has been 15 days, will my daughter be called up for a next cycle of chemo and oncologist have already stated that there’s nothing can be done for her.” | |
| “ Most patients come from very far away districts.. so most of the time, we are still not started palliative care in other district.. and patients are from those areas they want to go home as soon as possible, so we are unable to provide that kind of care to them... second is they let go.. they continue to ask for aggressive treatment, because they will have somebody in the family telling them that no no you have to go for this and they keep going back to parent department and they keep asking for some more.. and then the parent department they are like ok you take one more radiation.. and like this it keeps going on and on.. another barrier which I see is that I wouldn't say the barrier but it sometimes depends on the treatment like they need or they look or explore for alternative medicine.. and then in this whole chase to get cured, sometimes they try various things and sometimes the main core part of the care is neglected.. they don't want to give morphine when the patient is in pain because they feel that the other thing is taking care of it.. and generally among educated people I have seen that palliative care is almost like giving up for them.. I have seen a lot of educated people think that like they stop fighting.. Because we get lot of rich patients in home care.. very very wealthy people are referred to our home care people.. and the biggest challenge for them is to make them understand to stop unnecessary treatment which is not helping the patient.. and that carries a very negative connotation..” | |
| “ You see basically you need to accept the reality and see the reality of the situation like what kind of patients you are treating in India.. unfortunately almost 1/3 or 50% are coming with advanced cancers and who are not curable.. so you see blindly chasing cancer directed treatments and giving them unnecessary side effects without any benefit in survival and lot of families going bankrupt in the process because they sell their properties for treatment.. it is not only the patient, but the whole family as a unit needs to be looked into.. when we see this patient, because, what I ask myself when I see my patient - so is he going to benefit from this form of intervention? and what is its impact on the family and their caretakers? and everything needs to be taken care of as a unit.. So if you look from that angle, the picture is very clear as to where we need to stop the treatment and go for palliation.. and the other is the quality of life.. Where most of the symptoms are not addressed.. but if you have a human approach and see these patients, you see a lot of these patients will benefit from this.. not only drugs and surgeries but there is a human touch, caring and palliation and that should be integrated properly..” | |
| “ So it is variable.. so you cannot generalize like that… and it varies from patient to patient.. like if you see cancer patients, when they have almost tried everything they might be of an understanding that an aggressive management might not be helpful and they might understand that the palliative care is more important even without our much communication… but in other patients whom the life expectancy or the younger patients, they might need to communicate more and make them understand what will be good for the patient..” | |
| **Subtheme 1.c: Lack of family training on how to fulfill caregiving responsibilities** | |
| “ I’ll say actually it’s easier in India sometimes because the family can be a huge support. Collective autonomy can be a problem but sometimes it's it’s needed in our country and so collective autonomy and the responsibility, family taking it up, joint family, support system, caregivers, health services, you have people; you at least have some body look after. My own grandmother who lives in the US is in a facility..not because she cannot, she has, my own..my uncle and aunt who are who are there and they’re elderly, they are at home too. But the system allows that because now she has come to the stage where she has to go into an institute or facility which would not have happened over here. I am not saying that there is anything wrong. Tomorrow, I am be in a facility and I have nothing and I’m I’m only trying to and that's why I like what Dr SB (cue- national champion) always says ‘Create the world you want to die in’. It’s one of my favourite phrases, so, ‘Create the world you want to die in’ is what I am looking forward to doing but and I would not mind being in a facility myself because I don't think I’ll have too much of a choice [laughing loud] maybe decades down the line, but, I am not talking it’s wrong about being in a facility but…we do have a lot of strengths but we are not utilising it enough. And in AP where I work or here where I work, where I see the system falling apart because elderly people are at home and children are abroad and it is excruciating for both sides. So, unless we have a system in place which looks after them and we are growing old. Our geriatric population is increasing. We have so many challenges. We cannot expect the family to look after everyone. We can only..that can only be an added support. We have to start growing out of box in thinking; that it’s not wrong to be looked after in a in a facility and having family support with the facility would probably be a very nice thing. And which we have the resources actually and you also have these lovely, you know, vol..volunteer tourism and things like that which can happen in India. So, it could be a nice thing to take forward.” | |
| “I, I don’t think there is a big difference but it all depends on the cultural thing, that’s all. People we get in our hospice we are not dealing with patients who are very rich. You can see a private hospital set-up, it is different and it is filled up with all the patients who are well off, you know. They have a good education, they…they maintain a good privacy. It’s quite different from the patients who are with a very poor background. Our challenge in our palliative care, wherever it is, mostly we deal with the patients who are the worst cases kind, you know, they don’t know how even a proper hygiene. Family doesn’t know it’s important to take a proper brushing every day, it’s important to take bath every day. They don’t, they don’t feel like that… So, teaching these kind of patients betterment is showing a good improvement.” | |
| “Well I think it is difficult.. because it is such a wide area isn't it?.. mainly cancer you need adequate staff, you need adequate volunteers and staff.. in the sense qualified staff I mean.. like doctors to implement the treatment and to give the treatment.. for eg. if you go to a patient who is totally bedridden with cancer and who can't eat or drink, you have to teach them and the relatives on how to put a drip you know under the skin.. that sort of a thing.. and how to do an enema… and how to do a manual evacuation of the rectum.. all this you need particular staff for it.. so I would say yes, all these are very important… yes..” | |
| **Theme 2: Family caregivers in India may face an overwhelming tradeoff between caregiving and financial and time demands related to work** | |
| “Most of the patients family members don't look upon the patient because of financial reasons.. and mostly the family members go to the work in the morning.. sometimes the patient will be alone at home nobody to take care.. that is the biggest challenge to the attendants.. because of the financial reasons.” | |
| “Mostly an idea that a fellow is destined to die, so let him die.. why make an effort to spend time and money on this patient.. so it is called abandonment.. so you are not physically abandoning but you are abandoning the treatment.. so the person is physically in your family but you are asking the patient to endure it and die rather than taxing others time, energy and funds.. so this is what we find many a times that happens.. so if you do a phone contact you can overcome that.. so you have to proactively call.. why have you not come? how is the patient? is there pain for the patient? are you offering something for the pain? who is prescribing? do you have enough medication? you should keep doing that.. and then that will become a solution.. so they know whom to call now..” | |
| “Yes.. sometime I told you na.. I mean sometimes they have.. because of not so very cordial relationship within the family.. or people believe that they are looking after that and doing a lot and other children are not doing.. and maybe the family is busy with their own work.. the daughter in law some working somewhere, daughter is working somewhere and nobody is at home all the time.. so they leave it to the caregiver who is untrained and unmotivated..” | |
| “I don't know whether if Doctor 3, Site B had given you some insights. sometimes you feel very unwelcome.. like looking at the patient we know that there is a need.. but with lot of reasons you know like family dynamics, the family will not be so keen.. first time it is ok.. but second or third time even when we call people you will not find very good receipt or response from them.. and then you give them advice, medication, the time dosage, frequency everything.. then they are not adhered to.. many times na when you count the tablets of morphine they are having, you know that they are not giving them as per their dose..” | |
| “like that, ten days ago I went to one...with… one home they didn’t… lift the phone we searched for one hour for the house then we got the house. Then, they literally left the patient and vanished in the same house. We helped… the patient with told them how to feed the patient and after that they vanished. So, from that context I know they need a caretaker rather than a home homecare physician. So immediately I told VA ma’am and NR (cue- team member for PC-PAICE project). See this is three hours we spent with for one patient really they need home care” | |
| “I don't know.. here basically the social support sometimes is not very good.. so they may not bring the patient on time.. that is one thing we are facing.. second thing is that we give the medicines and if the patient is not educated enough to take it and you educate the care taker to give it, they will not give it.. if they won't give it properly, they won't get the pain relief.. whatever it is the symptom relief won't be there and they will come back again saying that you have given the medicine but I am not getting relief from the thing.. so then you go deep into it then you will understand that they are not compliant with the medicine and that the patient or the care taker or other things.. or sometimes the patients are left alone without anybody in the house, so some of these problems as such they are not unhappy with the palliative care.. and one more thing is that suppose you admit them in the hospice, some of them don't want to leave the hospice.. they want to remain there only because there is someone to take care of them.. even the attendants also feel very happy because they need not take care of them everyday at home no? so it is very difficult to handle a sick patient at home everyday so people need a kind of a respite care and other things.. so these are some of the things.  R2: And within the families, like is there any conflicts which affects you to deliver care?  I: Sometimes yes.. They won't tell.. they won't tell the truth to the patients.. What is that you call?  that is collusion.. All these things are very common and sometimes you must also be facing all this these things.. so they are not very new to you.. When they enter also with their eyes they say no no no don't tell the patient, those kind of a things.. but when they come to cancer hospitals they know and patient must be knowing.. They won't reveal it.. Very gently we convey them and we usually tell them the truth in the sense we tell them the truth..” | |
| “ I’ll say actually it’s easier in India sometimes because the family can be a huge support. Collective autonomy can be a problem but sometimes it's it’s needed in our country and so collective autonomy and the responsibility, family taking it up, joint family, support system, caregivers, health services, you have people; you at least have some body look after. My own grandmother who lives in the US is in a facility..not because she cannot, she has, my own..my uncle and aunt who are who are there and they’re elderly, they are at home too. But the system allows that because now she has come to the stage where she has to go into an institute or facility which would not have happened over here. I am not saying that there is anything wrong. Tomorrow, I am be in a facility and I have nothing and I’m I’m only trying to and that's why I like what Dr SB (cue- national champion) always says ‘Create the world you want to die in’. It’s one of my favourite phrases, so, ‘Create the world you want to die in’ is what I am looking forward to doing but and I would not mind being in a facility myself because I don't think I’ll have too much of a choice [laughing loud] maybe decades down the line, but, I am not talking it’s wrong about being in a facility but…we do have a lot of strengths but we are not utilising it enough. And in AP where I work or here where I work, where I see the system falling apart because elderly people are at home and children are abroad and it is excruciating for both sides. So, unless we have a system in place which looks after them and we are growing old. Our geriatric population is increasing. We have so many challenges. We cannot expect the family to look after everyone. We can only..that can only be an added support. We have to start growing out of box in thinking; that it’s not wrong to be looked after in a in a facility and having family support with the facility would probably be a very nice thing. And which we have the resources actually and you also have these lovely, you know, vol..volunteer tourism and things like that which can happen in India. So, it could be a nice thing to take forward.” | |
| The patient and the family members think that it is a sin on them to let the patients suffer and die in a painful way.. and if they don't take care of the patient then they have done some mistake.. and he is bearing that due to that sin.. | |
| **Subtheme 3.a: Adopting a family care model to train and empower the caregivers** | |
| “Since we have a palliative care family care model, so we train one family person to look after the patient after the discharge… even the dressing and heavy bleeding.. if it is started at home, so what could be done? so one person is trained by that way..” | |
| “yes villages mainly.. that is where the problem is.. so we do counselling and then do wound dressing for the patient and then teach the family members also how to do wound dressing.. and fluids also.. so if the patient or the family is unable to come to the hospital, then we do all the counselling there and explain everything there itself.. social reasons and everything.. we do counselling for the whole family on what to do and what not to do..” | |
| “Everyday basis, we try to counsel the ladies as well as the patient that maintain hygiene condition. We need to think of the patient. If we think of patient, we should not say ‘no’ to him, maybe this is his wish...maybe it’s his last wish, so, we should not deny his wish. On the other hand, we talk about the wife’s concerns. We tell her it’s not contagious disease. Nonetheless, concerns like malodor, untidy feeling can be taken care of by maintaining hygiene and covering the cancerous/untidy part. If the wife takes care of such things, then it can be possible. If there is so much love between the two, then that should be expressed also. Perhaps, patient needs that only now, i.e. wife’s love, so she should know how to take care of that. For example, if this happens with our own child, then would you hate him? No, you won’t, so, at times you can think like of him as your child and take him in your lap. We are dealing with such cases on day-to-day basis but we don’t have anything concrete or streamlined.” | |
| **Subtheme 3.b: Raising acceptance of PC through social workers, volunteers, and community networks** | |
| **“**So what happens is you know, the first thing when the patient comes, when we take the admission, the first thing is the social worker or the counselors sits with all the family members to meet them, to see what they understand and what they don't understand… ok what are their expectations.. what we can do for them.. if something is there like their expectation is the ICU care, so we told that, ‘we don't provide ICU care and we will not give it.. and we will see if you require ICU care and that is a necessity then we will definitely advise you… but we are not able to provide you.. and where you can go…’ that option we are given.. so they are not left with some unanswered questions.. so continuous talks with the social worker with these caregivers, slowly slowly they understand… so what we do is, once the patient is symptomatically better, we immediately discharge this patient and say stay at home.. we train the caregiver in caring for the patient.. how to turn the position, how to do the feeding, medicines, wound management.. even we train the caregiver on the wound management also.. so they are doing dressing at home… so they know that the patient can be discharged.. so they are seeing people being discharged.. so now that stigma is going down.. but for a few people who doesn't know anything, they just hear some and they will still have it..” | |
| “I think that these are some standard problems people have, the first thing would be that they would, they might not want us come home because they might be living in an area where they are renting a house and if they know, the owners know that child has cancer or the adult patient also; if they have cancer, they might ask them to vacate the house. People are unduly curious about what is happening, who are they, kind of thing, so our team tells them that we would not wear aprons, we would not bring medical equipment visibly. We would park our van like half a kilometre away sometimes and we would walk and you can tell them that we are friends of someone that you know, kind of things. So, lot of negotiation goes. Most of times we are successful but sometimes we are not. And they might..such patients, might offer for hospice care or they might want to come to OPD. Sometimes....the other issues are there might be, I mean, it is always difficult, right, going for home care. In the hospital, you are the boss. They are coming to your room, but they now you are going to their house [laughs] but..the feeling is different. But over a period of time I think you get used to it and the families I have visited were really really nice like you know that they are very very poor, they can’t afford that milk but they make the tea, the coffee, they give it to you. They get those cool drinks and they give it to you. They want to feed you, so, yeah, I mean, the families have been really receptive that way. And the paediatric home care, there is an exclusive van right now and we have enrolled all the curative patients also in the paediatrics palliative care who live in City-A. So, we have been able to get back around and children who have left against medical advice, absconded from treatment; we have been able to go back, convince them. The social workers were very very they are very experienced ones. So, they were kind of able to break the barriers and convince them to come back, so, yeah.” | |
| “So you need to do it proactively.. if you don't do it proactively then they won't bother.. why worry about it.. If you can offer them that - yes I can come home, I will see your patient, I will train your family on how to give medication, on how to take care of colostomy or a fungating wound and I will give you the free medication as well for the patient or something like that they are very happy with that.. so you need to overcome the barrier proactively.. then they will motivate somebody else and then somebody else and again somebody else in the same city or town.. 98% of our reference are actually from word of mouth.. I went to that hospital and I died very peacefully.. so you can also take them there.. so we get reference now only for palliative care.. We don't see them.. they go directly to palliative care..” | |
| “For example, if we take a paediatrics case, the kid will be affected and mother is going to take care of him them. Father will be like questioning, ‘It's all because of your fault, look at his suffering’, like this. So, he..it's, it's none of their fault. It's because of the developmental problem in the body of the boy, so, he is suffering. So, that thing they won’t understand..and if we deliver it in a proper way also it's not...it's does not go into their mind because I am just single doctor who is explaining in a proper way but he listens to all the surrounding area, his relatives etc. etc. and they will think that you should have took him to that hospital, you could have took him to that hospital. There is not that disease, it is this, this is all because of your wife, this is all because of you, all these things. So, it will be very difficult for one person to clarify the thing. From the..it should be also coming from other end. So that the person should believe what’s happening in..in for sure, that this is the process which happens because we go through the medical things and we we study all these things. So, the outer persons are not very appropriate about this knowledge. So, if a doctor speaks out, there will be multiple doctors who will be..not delivering it in a proper way. So, that will also give a bias thing and….” | |
| “Yeah neighbourhood network palliative care is like, we are training.. I mean we have put up a notice.. initially those who are interested in volunteering in our palliative care, they need to take the 3 day course first.. so when they come to us, we make them aware about what palliative care is all about and how important that is in our community.. and then how they could identify the needy people about the palliative care system and how they would assess the people who need palliative care and bring them to us.. so suppose a person in the locality of a small village, they could go around or they could just identify even by mouth.. when they talk about the awareness, then the next door neighbour would come and tell that, ‘you know, there is a palliative care network in City F.. these are the things that they do.. so you don't have to go through all the suffering all alone.. and that they will be much of help’.. I mean so those kind of awareness to the neighbours and other people, and then some of their relatives would be going.. so that will be passed on to the others.. so they will be brought to us by the volunteers you know and they would get the help..” | |
| “That is the reality… so people are very I mean palliative care and end of Life Care is one thing.. but volunteers only those will come who have experienced cancer in the sense.. but then there are a lot of volunteers, who come and who assist us in fundraising activities.. and also we have a free kitchen in our hospice where we give free food for the patient and their caregivers.. so volunteers come and donate rice and groceries etc.. and some volunteers want to do counselling but by and large after coming there once or twice, they are not able to take it.. so volunteering in a hospice is a different and difficult thing..” | |
| “so we asked them what motivated you to do this.. we know a lot of pathologists have moved to palliative care.. a lot of radiation oncologist have moved to palliative care.. some surgeons have moved to palliative care.. and the initiation is that they had somebody in the family who actually suffered very badly when they were terminally ill and nobody was there to guide them as to where to go for xyz.. so that is the actual motivation.. so that is the personal tragedy which motivated them.. so that is important.. so that is a good point to rally around.. they will now start to relat to what they could have done back to the family member.. so if it was somebody very close to them like mother or father or daughter or son all the more reason they will become more passionate about it..” | |
| **Subtheme 3.c: Responding to the family caregiver’s requests** | |
| **“**Yeah those things are there in a huge way because cancer is sometimes they think it is contagious.. these people.. that is not contagious and that is why it affects our services.. and the neighbours don't like an ambulance coming to the house.. so sometimes the ambulance has to be parked far away and then many times often, the patients and the relatives don't tell their neighbours that somebody is down with cancer.. so they say ‘no no.. you have to come in secret.. don't come when they are seeing..’.. so our people face this problem of explaining to the neighbors and then trying to convince this family that there is nothing wrong if we come and look up to you.. all that is there.. a lot of those problems are there” | |
| **“**It is actually you know like, when we really meet the patient in person, and the patient you know sometimes they do want to talk to us.. you know like they talk about what they know about the disease process.. initially we just want to know how far they know about it, so we have to know how far they know about it.. and then we will get some sort of a privacy you know, sometimes the family relatives or the family members they come just before handing over the patient and they just say that ‘the patient does not know about the diagnosis and the actual situation.. and so we would like, you're the doctors to actually not talk about it’..” | |
| **“**Yeah and when the patient asks us without the family consent you know. that we may not be in a position to disclose all the details to the patient.. because that may cause a problem between the patient and the family members.. because they are the people who are caring for the patient 24 hours.. so we have to win their confidence first.. so we go to the family members and make them understand that there is a need for communication and that we don't have to say that the patient is having cancer or it has even metastized to so many parts… and I mean we are not destroying Hope, but we are giving an information that this is a disease that which cannot be cured so easily and that we have to proceed slowly and then see how it is evolving and slowly slowly, bit by bit, we have to give the information.. not all at once.. so like that, in their presence, we start communicating with the patient.. and also we use another term for cancer.. like instead of saying that cancer directly onto their face, we modify the terms and make the patient prepared to accept the worst news.. so like that we move on.. this may not be possible on a one time visit and we will have to make multiple visits..” | |
| **“**We usually counsel the family members.. that we make them sit and try to explain to them.. like we try to find out the reasons like why they don't want us to disclose it to the patient.. they usually give only one reason... like he will panic and that he will going to depression… so we just try to explain to them, that it is not just like that.. even they are roaming to so many hospitals and from one department to another department undergoing investigations.. so they also like to know what is happening to them.. and by this time they might have known that they are suffering from a very serious problem..” | |
| “Formally whenever we found a situation of Collusion we didn't try to clarify that.. now after taking up this initiative, we have started exploring those issues of Collusion and are taking proactive steps to resolve such collusion.” | |
| **Subtheme 3.d: Providing material resources such as financial relief and mental health care for the caregiver** | |
| “Yes yes.. I am the coordinator for the student group, because i was and I am basically a teacher.. so I was assigned this.. so we have this NSS - the national service scheme groups in colleges.. so they have a faculty coordinator from the college.. so we actually, I take their help and it starts like this.. we give an awareness class to them in the beginning of the year.. we give awareness classes for around 100 students.. from there around 10 students or sometimes 5 students or 20 students, who ever take interest in palliative care from each Institution, they come to us and we give them 1 day workshop.. that is for the students.. and advanced training that is the 2 day volunteer workshop also is conducted in case the students are willing to work independently in their own area.. so this is the first step.. the second step is if the colleges are far away from our institution, then we link them with a Primary Health Care Centre or the Taluk hospital or whichever government program which has the palliative care unit.. so they start going for home care and you know patient care in their own area.. very few students are there who actually take up this near their home.. also so we encourage that that the student can be a regular caregiver to the patients in their own area.. but that is only a few cases that we get that kind of a response… but otherwise they like to work in a group.. even the parents also only allow that.. and the institutions also supports that.. so they conduct and they do home care.. they identify the needs, the social needs mainly.. so they conduct rehabilitation camps where they teach the patient or the family to make some products.. and there is a college who even supports selling those products in the campus.. like they have been trained to make paper pens and this campus, the students recommended to the principal, this particular campus, they banned the usage of plastic pens and the other college now uses only paper pens made by these patients… they go even after that extent you know… it is their creativity and their own freedom that we give them… and then it comes to our own group, who also want to work with us.. they come to us on holidays and Sundays and we arrange home care.. and mainly you know the cleaning of the houses.. like the coming week, we have this psychiatric patient.. like the house of a psychiatric patient.. we get the cooperation from the government.. like whoever the counselor or the ASHA, they will be there with us.. the patient will also give the consent.. then we go there and we clean the house… and you know this kind of activities.. then there is the geriatric patient or the bedridden patients.. they just love the company of this children.. they just go and sit there and they talk... so we actually identify the need, like which kind of patients actually accept the students and then the regular kind of.. And then some of them form a relationship.. and they like keep a healthy relation and they try to keep the contact on a regular basis.. there are young patients who like their company.. so they arrange programs for them.. so some of them, they invite these patients to their college campus in their programs.. it is just that because they are youngsters, we give them the freedom of you know arranging or organising things to them… we just monitor them that is all..” | |
| “Ok.. this is not kind of a newly we started, but these are going on for the last 15 to 20 years.. there are a few additions and there are a few deletions.. so we have a meditation.. so it is not a hard core meditation.. It is simple relaxation techniques.. some patients just come and sit and they enjoy watching other people doing the stuff… there are entertainment programs happening twice a week.. and today was one in the morning and will be next Saturday.. so it is just not an entertainment program, like if anybody is not well in the house, the other person's life gets affected.. no TV, no party, no outside dinner, no friends, don't talk loudly.. all those things happen.. and now this patients caregiver is here for the patient 24*7, and they do not have their personal life.. so that balancing becomes difficult.. and this patient is eventually going to die, so what is he left with? Nothing… ok, so how do you now balance that thing?.. so we started this entertainment program.. so we found relatives and told them that ‘we will take care of your patient.. you go and take part’.. and those patients who can also take part, they also go there.. first initial 2-3 times what happens is that, they come back to check on their patients… they feel very guilty about it.. but they slowly realise that ‘I am watching this program and halfway I am going.. I am feeling much relaxed and much happy’.. and seeing that happy face, the patient feels better… and their performance as a caregiver has also improved… so we are somewhere going to do some study on the same, like how you know show that results.. so in that case what happens is, this patients caregivers slowly start believing that by doing that thing like, ‘ok I am attending the entertainment program of singing, dancing or whatever it is.. but you know I should not feel guilty.. I can balance both the things’.. when you try to tell them, they don't understand and they will say, ‘what do you know about it? it is so difficult’.. but when they actually do it no, then they are able to do that thing and when they get discharged at home then it helps there… so that is what I was saying, then this activity actually gets converted into therapy… so these are the things…  and some patients are from agricultural backgrounds.. so we have quite a huge garden over here.. so some people who want to do some gardening, I think we have some horticultural activities also.. so we arrange sometimes, so that you know it helps them to feel that you know, ‘I am not completely useless.. I am still able to do what I used to do’.. so they find it solace you know.. just touching the soil also.. so these kind of activities are there.. then Handicrafts are there.. This reading and Library facilities are also there.. all this..  what we want to say is that, everybody has some activity that they do at home in their leisure time or something.. but now they don't have the confidence or energy to do the same thing.. so we are introducing the small activities to them.. so once they start doing it and they are able to do it, like ‘see if you can do this and try what you used to do before’, you may be surprised that you may be able to do those things.. ok and that is how they start.. so that is when the activity actually gets turned into therapy… and they can get back to their normal activities.. not completely but partially.. so even these activities that we do over here point them there..Other things like celebrations are there.. one of the important things, now you had that Prasad from the temple called City AM ok.. so you must be knowing that this is a you know, the most famous temple in State 6.. and lots of people, every year or once in a year go there on walking from their houses.. they don't go by car or something.. and there is a big Yatra kind of a thing.. it is called ashadi Ekadashi.. so they go walking and they have that Holy Basil tree on their head.. and they will do lots of singing bhajans and all those things.. but there is no superstition in this kind of activities.. and now these people are doing this for ages.. every year they go walking from their home.. like they travel 300-400 kms by walking.. and now they cannot do anything.. they are not able to go because they are too sick.. the relatives cannot take them or the relatives are not able to go because they have a patient here.. so we arrange that small procession or Yatra here itself.. so it starts from the auditorium and it goes to each and every ward with all those patients who can walk, for those who can come on the wheelchair, the relatives comes and around the centre they just go out of the gate and come back again.. so getting that little bit feeling of ‘ok’.. this kind of things.. so these are also actually becomes the diversion activities.. and ultimately it is quite comforting for them..” | |
| “Collusion is quite high, adult and paediatric, very very high but I think over a period of time..by talking to them, I think most of the families do get convinced but then they are definitely few who would just say no. The cancer stigma is also very much present. People think that cancer is a communicable. They are worried that their children might get it, kind of things. So, repeated explaining that it is not communicable, it is not hereditary and things like that. So that has to happen quite a lot here.. Surprisingly, they are not told that cancer treatment is not available that is why we are sending to palliative care. Instead the oncologists are very busy and they just write down room number 34. So, they have no clue. They think we are other oncologist and they come to ask to us. So, then we start explaining that, we are not oncologists, we will give you supportive care, kind of things. So, I think most of the times they come back because..we are very good at managing their symptoms so I think that is the reason why they come back. And, then we also give them a good ear. We also listen to their problems, so, yeah.” | |
| “Take death... the availability of morphine is a huge thing.. because morphine is not available in many hospitals because of the NDPS act being so strict.. so we ensure that morphine is easily available.. one of our important work is to ensure the availability of morphine for all these patients so that pain can be controlled.. so if you ask me one thing peaceful death.. It has to be peaceful.. it has to be comfortable.. there should be no pain.. and most importantly the person should die in dignity.. that is the main thing.. so we have done a lot of things and a lot more needs to be done to ensure that these people breathe their last in dignity.. preparing the family for death is another thing.. that's very important because what happens is the doctors or the oncologists who treat them, they don't tell them that your treatment is over.. they don't tell them.. they just refer them to us.. it is our doctors and nurses and our counselors who have to break the news to them most of the times, that this is going to happen, that there is no further treatment and that what we are giving is palliative and end of Life Care.. so if you ask me one thing, one very specific thing that we have improved on, it is to ensure peaceful death and also preparing the family for the death.. and how do we measure it? most of the times we measure it by the feedback we get.. because in our hospice and home care we have almost weekly meeting with the caregivers.. the caregivers meeting is a big thing for us and during that time we ask them, was the death peaceful? was our staff there to ensure that the relatives and the caregivers were prepared that this is going to happen?” | |
|  |  |
